# An Assessment of Semaglutide Safety Based on Real World Data: From Popularity to Spontaneous Reporting in EudraVigilance Database

**DOI:** 10.3390/biomedicines12051124

**Published:** 2024-05-18

**Authors:** Anca Butuca, Carmen Maximiliana Dobrea, Anca Maria Arseniu, Adina Frum, Adriana Aurelia Chis, Luca Liviu Rus, Steliana Ghibu, Anca Maria Juncan, Andrei Catalin Muntean, Antonina Evelina Lazăr, Felicia Gabriela Gligor, Claudiu Morgovan, Andreea Loredana Vonica-Tincu

**Affiliations:** 1 Preclinical Department, Faculty of Medicine, “Lucian Blaga” University of Sibiu, 2A Lucian Blaga St., 550169 Sibiu, Romania; anca.butuca@ulbsibiu.ro (A.B.); carmen.dobrea@ulbsibiu.ro (C.M.D.); anca.arseniu@ulbsibiu.ro (A.M.A.); adina.frum@ulbsibiu.ro (A.F.); adriana.chis@ulbsibiu.ro (A.A.C.); liviu.rus@ulbsibiu.ro (L.L.R.); ancamaria.juncan@ulbsibiu.ro (A.M.J.); andreicatalin.muntean@ulbsibiu.ro (A.C.M.); felicia.gligor@ulbsibiu.ro (F.G.G.); claudiu.morgovan@ulbsibiu.ro (C.M.); loredana.vonica@ulbsibiu.ro (A.L.V.-T.); 2 Department of Pharmacology, Physiology and Pathophysiology, Faculty of Pharmacy, “Iuliu Hatieganu” University of Medicine and Pharmacy, 400012 Cluj-Napoca, Romania; 3 National Institute of Research and Development for Electrochemistry and Condensed Matter, 144 Dr. A. P. Podeanu, 300569 Timisoara, Romania; antonina_pop@yahoo.com

**Keywords:** semaglutide, obesity, weight loss, overdose, underdose, off-label use, EudraVigilance, GLP-1

## Abstract

Some glucagon-like peptide-1 receptor agonists (GLP-1 RAs), first used in the treatment of type 2 diabetes mellitus (T2DM), have been approved for the treatment of obesity in patients with or without T2DM (liraglutide—LIR, semaglutide—SEM, and tirzepatide—TIR). Social media had an important influence on the off-label use of GLP-1 RAs for obesity, especially for SEM. We analyzed the Google queries related to SEM to assess people’s interest in this drug. We also investigated the occurrence of adverse drug reactions (ADRs) by searching the EudraVigilance database (EV) for Individual Case Safety Reports (ICSRs) that reported SEM as the suspected drug and performed a descriptive and a disproportionality analysis. The data obtained for SEM were compared to other GLP-1 RAs. SEM had the highest proportions of searches on Google associated with the term “weight loss” and presented the lowest number of severe ADRs, but it also had the highest number of ICSRs reported in EV. Even though no unexpected safety issues have been reported for it until now, SEM has a hi3gh tendency for overdose reports. The most frequent off-label use was reported for SEM and TIR. In order to lower the risks of ADRs, the off-label use should be reduced and carefully monitored.

## 1. Introduction

Obesity is considered one of the most common metabolic diseases, often associated with an elevated risk of developing type 2 diabetes mellitus (T2DM), non-alcoholic fatty liver disease, and cardiovascular disorders like hypertension and heart failure with preserved ejection fraction, etc., thus reducing the life expectancy of patients [1,2,3,4]. Recent studies show that obesity increases the number of hospitalizations, the need for mechanical ventilation and the incidence of death in patients with SARS-CoV-2 [5]. According to the World Health Organization (WHO), in 2022, 2.5 billion adults were overweight, and of them, 890 million were obese (~12.5%). The same report pointed out that the number of overweight children under the age of five was 37 million. In the 5 to 19 years group, over 390 million children and teenagers were overweight, of which 160 million were obese [6].

Known as one of the biggest challenges of modern society at a global level, the fight against obesity has been declared a real public health emergency [1,5]. On the other hand, diabetes is considered one of the most widespread worldwide medical conditions and T2DM is the most common form [7], with both acute and chronic consequences which decrease the quality of life, reduce life expectancy and increase the mortality rate [8].

One of the global goals of the WHO is to stop the increase in diabetes and obesity by 2025 [6]. Unfortunately, until now, lifestyle changes in terms of daily diet and physical exercise have often proved insufficient in achieving significant weight loss [7,9]. The connection between obesity and diabetes is very close, so a large part of obese people are affected by diabetes or have a very high risk of developing T2DM in a very short period, and many patients with diabetes, especially those with T2DM, start to gain weight, soon becoming overweight or obese [10].

Significant evidence attests that an effective improvement of insulin sensitivity and simultaneous reduction of the risk of diabetes associated with obesity can be achieved through weight loss [10]. Over time, several molecules have been administered for the treatment of obesity, but their limited efficacy and/or adverse reactions led to the limitation of their use or even their withdrawal from the market (e.g., sibutramine, amfepramone, rimonabant, benfluorex, dexfenfluramine etc.) [5,11,12,13]. In this context, the approval of new drugs with adequate efficiency and safety in obesity treatment has been sought. Thus, the approval of the first glucagon-like peptide-1 (GLP-1) receptors agonist (GLP-1 RAs) in the treatment of T2DM, with structures similar to endogenous hormones, opened a new era in promoting weight loss and improving health outcomes in obese people, including those with comorbidities [14].

**Figure 1:**
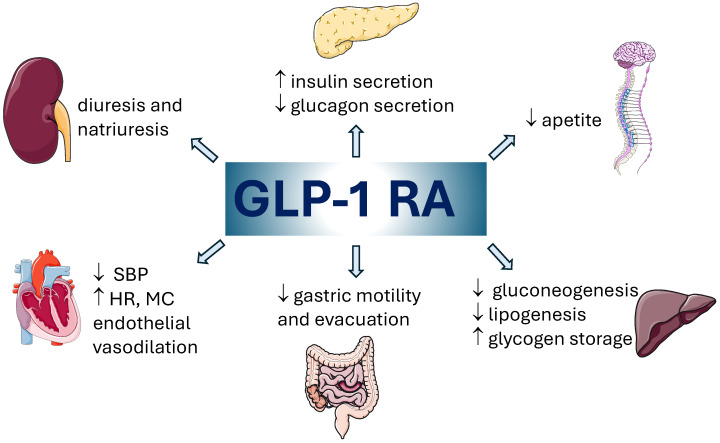
The main biological activities of GLP-1 RAs [25]. SBP—systolic blood pressure; HR—heart rate; MC—myocardial contractility; ↑—increase; ↓—decrease.

GLP-1 and glucose-dependent insulinotropic polypeptide (GIP) are two of the main incretin peptide hormones excreted in the intestinal tract [15], responsible for increasing the secretion of insulin after eating food and also inhibiting the secretion of glucagon [16,17,18,19]. GLP-1 reduces gastrointestinal motility, which in turn extends the period when nutrients might be absorbed. It also determines the feeling of satiety, enhances resting metabolic rate, and decreases free fatty acid concentrations in plasma [1]. Because GLP-1 receptors are expressed in extra-pancreatic tissues, GLP-1 presents a lot of extra-pancreatic effects: delayed gastric emptying, appetite suppression, weight loss, glucose uptake in the muscles, decreased glucose production in the liver, cardiovascular protection, neuroprotection, renoprotection etc. [19,20,21,22]. These effects determined that the use of GLP-1 RAs has a high potential to reduce body weight and to be considered for the treatment of obesity [15] (Figure 1). GLP-1 RAs have been approved in the treatment of T2DM for improving HbA1c and for reducing the risk of major adverse cardiac events (MACE) in diabetes patients with cardiovascular risk [23,24].

Currently, there are several GLP-1 RAs approved worldwide, mainly for the treatment of diabetes. Exenatide (EXE) was the first GLP-1 RA in the world approved by the Food and Drug Administration (FDA), in 2005, for the treatment of T2DM, with two administrations per day before meals [26]. Later, the European Medicines Agency (EMA) (2009) and FDA (2010) approved the second agonist, liraglutide (LIR), as an adjuvant to diet associated with physical exercise in patients with T2DM. Compared to its predecessor, the latter presented a longer half-life with a greater effect on the reduction of HbA1C; at the same time, it showed cardiovascular benefits in addition to its effects of lowering blood sugar. In addition, it can be administered once a day regardless of meals. Moreover, the FDA (December 2014) and EMA (March 2015) approved LIR for the treatment of chronic weight management [26,27]. The manufacturer improved the short-acting time of EXE and a new formulation was approved in 2012 by the FDA. Compared to the old formulation, the prolonged-release suspension of EXE is administered once a week [28]. In 2014, the second GLP-1 RA with weekly administration, albiglutide (ALB), was approved by the FDA for the treatment of T2DM in patients who cannot reach glycemic goals. In 2018, the manufacturer withdrew ALB for commercial reasons [29,30]. Also in 2014, dulaglutide (DUL) was approved by the FDA. It had the advantage of reaching the therapeutic concentration faster than other GLP-1 RAs with weekly administration. Semaglutide (SEM) was launched in 2017 in injectable form, and it had an extended half-life of 7 days. In 2019 the oral form of SEM received approval from the FDA, thus being the first oral GLP-1 RA treatment for adults with T2DM [29,31]. Tirzepatide (TIR), a dual GLP-1 RA and GIP receptor agonist (GIP/RAs), is the latest one launched on the market, approved in 2022. GIP triggers glucose-dependent insulin excretion and is responsible for a larger fraction of the incretin effect than GLP-1. Depending on glycemic status, the glucagon secretion could be increased in normoglycemic or hypoglycemic patients (glucagonotropic effect) or inhibited in hyperglycemic patients (glucagonostatic effect) [22,32,33].

Preclinical studies have shown that GIP can decrease body weight by diminishing food intake and enhancing energy expenditure and, in combination with GLP-1 RAs, can have a greater lowering effect on blood glucose and body weight in patients [1,19]. Clinical studies have confirmed their effectiveness in weight loss and some of them (LIR, SEM, and TIR) have been approved for chronic weight management [23,24].

LIR was the first GLP-1 RA approved for weight loss in patients without a history of T2DM. In 2014, it was approved by the FDA in adult patients with a body mass index (BMI) larger than 30 kg/m^2^ on its own or with a BMI greater than 27 kg/m^2^ associated with at least one comorbidity related to weight, such as hypertension, diabetes, or dyslipidaemia [25,34]. SEM only received approval from the FDA for weight control in 2021 [34]. In the clinical studies carried out in this respect, SEM was superior in comparison to other long-acting GLP-1 RAs from the same class, namely EXE [35] and DUL [36]. Moreover, in addition to its beneficial effects in T2DM and in controlling body weight, it also showed a significant decrease in the rates of deaths due to cardiovascular issues, non-fatal myocardial infarction and non-fatal stroke in T2DM patients at risk of cardiovascular diseases [34]. Thus, LIR could reduce body weight by up to 9.2% (56 weeks) [37,38], SEM up to 17.4% (68 weeks) [39,40], and TIR up to 20.9% (72 weeks) [39]. Compared to LIR (16%), SEM leads to a higher reduction in caloric intake vs placebo (35%) and reduces the food cravings, which suggests different mechanisms of energy intake regulation [41].

Real-world data are a valuable resource for healthcare research [42]. The information within is obtained from a heterogenous population [43,44] and is collected by various methods such as electronic health records, pharmacovigilance databases, and search engines, contributing to revealing the existing clinical aspects [45]. Appropriate analytical techniques are necessary to extract reliable results from the extensive raw data [46]. Both the research community [47] and several medicines regulatory authorities [48] have shown great interest in this domain and are preoccupied with setting quality standards in the field [49]. Real-world data complement the results of long-established research methods such as clinical trials, enhancing the accuracy of evidence-based clinical profile [50]. SEM is an adequate candidate for a real-world data assessment, due to the large interest of the population for this molecule. Although intended for diabetic patients, many non-diabetic ones have used it for aesthetic body adjustments [7,51]. The off-label use of SEM for weight loss was promoted by social media and heavily influenced by famous public figures [47]. This intense media coverage led to numerous shortages of this drug with major consequences for patients with T2DM. Beyond these shortages, the incorrect and inadequate use of GLP-1 RAs can have major consequences on the health status of the population. Until now, various studies have shown the negative impact of these drugs, mainly because of gastrointestinal disorders (pancreatitis, nausea, vomiting, etc.), renal failure, liver injury, allergic reactions etc. [52,53,54,55]. This study aimed to identify public interest in searching for information about SEM online and also to analyze the secondary real-world data regarding the use of inadequate doses (overdose, underdose or incorrect dose) or even the off-label use of SEM. In this respect, after the analysis of data presented on the Google Trends Tool, a detailed analysis of the Individual Case Safety Reports (ICSRs) uploaded in EudraVigilance (the European adverse reaction reporting database) was carried out. The evaluation of SEM popularity and the safety profile was performed through comparison with other GLP-1 RAs (including ALB, which was withdrawn from the market).

## 2. Materials and Methods

### 2.1. Study Design

In January 2021, Google had 91% of the market share of online searches worldwide, representing the main search engine [56]. Thus, the present study started with an analysis of the popularity of search queries that were entered into Google Search. A relative search volume (RSV) is generated from Google Trends. The RSV does not provide the actual number of searches but presents data on a relative scale. The numbers represent search interest relative to the highest point on the chart for the selected region and time. A value of 100 is the peak popularity for the term, while a value of 50 means that the term is half as popular [57,58,59]. In the present study, the data obtained from the Google Trends Tool were analyzed considering peoples’ interest in searching for information about GLP-1 RAs. The terms used for comparison were the International Nonproprietary Names of each GLP-1 RA: “semaglutide”, “liraglutide”, “tirzepatide”, “albiglutide”, “dulaglutide”, “lixisenatide”, and “exenatide”. The comparison was performed worldwide, between December 2005 and March 2024. The interest score is presented on a scale of 0 to 100, where 100 indicates the highest level of popularity and 0 represents the least amount of interest. Subsequently, the popularity by region of each GLP-1 RA was analyzed. For each molecule, 100 points were allocated to the country with the highest popularity, and, to the other countries, the number of points was allotted proportional to the number of searches. Furthermore, we identified the first 25 related queries which were searched for by the same consumers who performed the searches for GLP-1 Ras, and we analyzed the frequency of terms related to “side effects” and “weight loss”.

The high popularity of SEM in the media could lead to self-medication and irrational or abusive consumption, potentially bringing on an increased number of ADRs, especially those which are severe or fatal. Thus, a retrospective pharmacovigilance analysis was performed based on the ICSRs uploaded in the EudraVigilance database until 31 March 2024 [60]. Firstly, a descriptive study of ICSRs reported for SEM was performed in comparison with other GLP-1 RAs (ALB, DUL, EXE, LIR, lixisenatide—LIX, TIR) or the entire group of all other GLP-1 RAs. ALB was withdrawn by the manufacturer because of commercial reasons, not for safety or efficiency reasons. In this context, we decided to use ALB for comparison, too. On the other hand, the ICSRs reported for the combination of LIR and degludec insulin were excluded from the present study. In the next step, a disproportionality analysis was performed to compare the reporting probability of ADRs for SEM with other GLP-1 RAs and with the entire group of all other GLP-1 RAs. No ethics approvals were required for the present study because no patients’ personal information was included in the ICSRs [61]. Healthcare or non-healthcare professionals filled out the reports from the European Economic Area (EEA) or non-EEA [62].

### 2.2. Materials

The chronologic data from the Google Trends Tool reported for SEM were compared to the series of the other GLP-1 RAs. Regarding ALB and LIX, the search interest compared to SEM represents <1%. Thus, both molecules were excluded from this analysis.

According to the Medical Dictionary for Regulatory Activities (MedDRA) hierarchy, many preferred terms (PTs) were used for reported ADRs. Each PT can describe “a symptom, sign, disease diagnosis, therapeutic indication, investigation, surgical or medical procedure, and medical social or family history characteristic”. In the next level, related PTs form the “High Level Terms” (HLTs) group, and related HLTs form “High Level Group Terms” (HLGTs). The final level for classification is represented by “System Organ Classes” (SOCs), with each SOC being formed from many related HLGTs [63]. At the moment, the total number of SOCs is 27.

In the present study, 4 HLTs were identified (modified dose, overdose, underdose, and off-label use) [62,64]. Thus, of a total of 35 Preferred terms (PTs), only 27 were identified in the ICSRs uploaded for the evaluated drugs (Table 1).

### 2.3. Data Analysis

A descriptive analysis of cases reported in patients treated with SEM was performed. The data were compared to other GLP-1 RAs. The descriptive analysis was structured by taking into account the general characteristics such as age, sex, category of reporters, geographic origin, and seriousness. In the next step, the distribution of ADRs by SOC was compared for SEM with all other GLP-1 RAs.

To evaluate the risk of incorrect dosage or off-label use, many PTs were identified for each of the 4 HLTs (categories of ADRs): modified dose (11), overdose (5), underdose (10), and off-label use (9) (Table 1). The total number of ADRs for each HLT was determined and compared between SEM and all other GLP-1 RAs. Subsequently, the outcome of ADRs grouped in HLTs was also compared. According to the EMA rules, the outcomes of cases are classified into 6 categories: (i) fatal, (ii) not recovered/not resolved (NR/NRS), (iii) recovered/resolved with sequelae, (iv) recovering/resolving, (v) recovered/resolved, (vi) not specified, (vii) unknown [65].

A disproportionality analysis was performed to evaluate the probability of reporting adverse reactions included in the four categories. According to EMA recommendations, the reporting odds ratios (RORs) and 95% confidence intervals (95% CI) were calculated for each evaluated HLT [66], according to a previously published protocol [67,68]. The disproportionate signal was obtained when the number of ADRs was ≥5 for each HLT and the ROR was statistically >1 (lower limit of 95% CI > 1) [66]. The data calculated for SEM were compared with each other GLP-1 RA and with the entire group of all other GLP-1 RAs.

## 3. Results

### 3.1. Analysis of Searching Google Popularity

**Figure 2:**
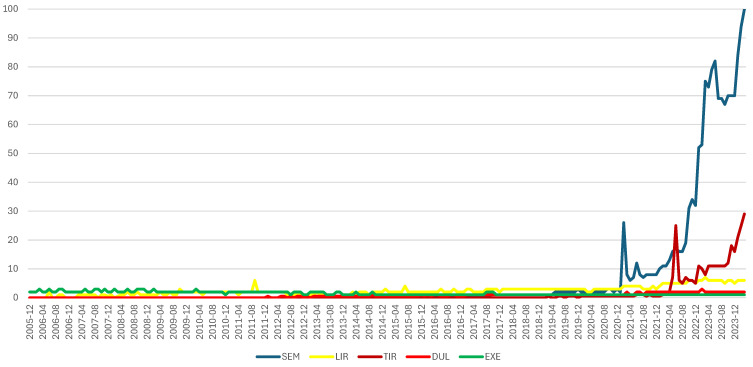
Comparison regarding the search interest related to GLP-1 RAs, according to Google Trends Tool (December 2005–March 2024) [69].

Based on the worldwide chronologic series obtained from Google Trends, starting with May 2019, the interest in SEM showed a constantly increasing tendency. Thus, for SEM, the highest level of interest was in March 2024. For other GLP-1 RAs the interest was lower than for SEM. For example, for LIR the highest number of searches was in June 2023 (~7% of total searches for SEM) and for DUL this was in February 2023 (~3% of the total searches for SEM). Since January 2022, the search interest has been increasing for TIR, the newest molecule approved on the market from the GLP-1 RA class. Thus, in March 2024, the inquiry proportion for TIR was about 29% of the total SEM searches (Figure 2). According to the same series, the search for ALB and LIX compared to SEM represents ~1%. Thus, both molecules were excluded from this analysis [46].

**Figure 3:**
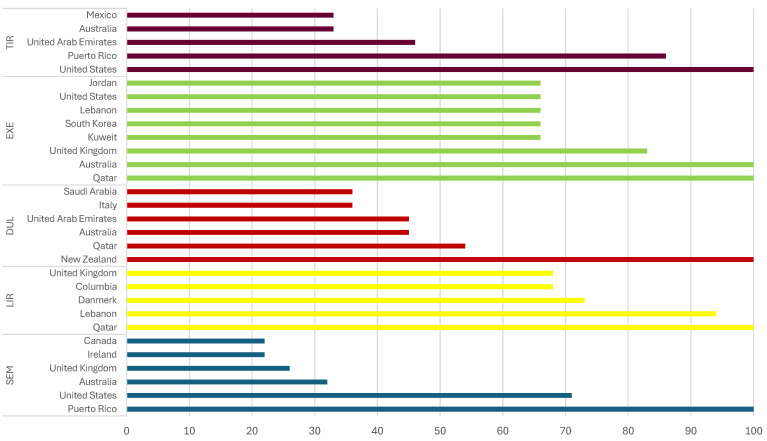
Comparison of interest in searching for information about GLP-1 RAs on Google by region [69].

The searches for SEM were more frequent in Puerto Rico (100), the United States (71), Australia (32), United Kingdom (26), Ireland and Canada (22). The highest level of popularity for LIR was in Qatar, for DUL in New Zealand, for EXE in Qatar and Australia, and for TIR in the United States (Figure 3) [69].

Consumers who searched for GLP-1 RAs also searched for other related queries. The most frequent term used by consumers who also searched for “semaglutide”, was “weight loss”. Thus “weight loss semaglutide” was the most frequent related query for SEM. Also, another two terms that were identified as related queries for SEM were: “semaglutide for weight loss” and “ozempic weight loss”. An interesting observation was in the TIR series, where “semaglutide weight loss” was one of the twenty-five most frequent terms associated with TIR queries. At the same time, people had a high interest in the side effects of SEM (“side effects semaglutide”) (Table 2). The queries related to weight loss were more frequent than for the side effects for all other GLP-1 RAs, except EXE [69].

### 3.2. Descriptive Analysis

#### 3.2.1. Analysis of ICSRs

**Figure 4:**
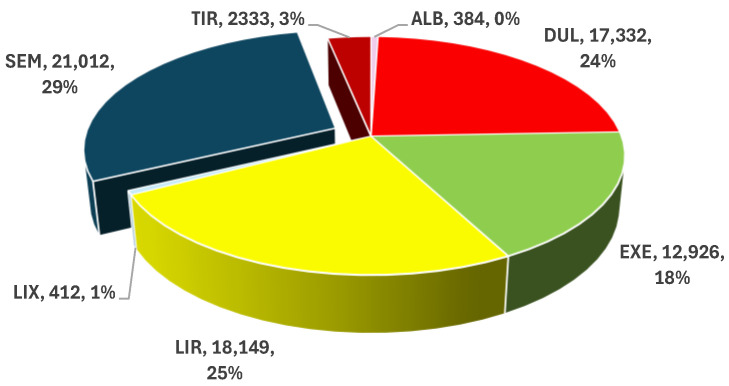
The shares of ICSRs that reported GLP-1 RAs. ALB—albiglutide; DUL—dulaglutide; EXE—exenatide; LIR—liraglutide; LIX—lixisenatide; SEM—semaglutide; TIR—tirzepatide.

From a total number of 72,548 ICSRs uploaded in EV until 31 March 2024 for the GLP-1 RAs analyzed, 21,012 ICSRs have been reported for SEM. SEM had the largest share (29.0%) of the total, followed by LIR (25.0%) and DUL (23.9%) (Figure 4).

**Figure 5:**
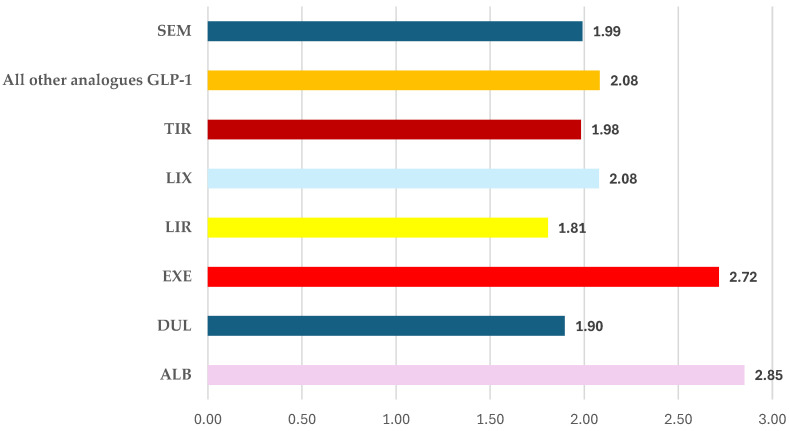
The proportion of ADRs reported from the total ICSRs. ALB—albiglutide; DUL—dulaglutide; EXE—exenatide; LIR—liraglutide; LIX—lixisenatide; SEM—semaglutide; TIR—tirzepatide.

The proportion of ADRs reported from total cases treated with SEM (1.99) is less than for the group of all other GLP-1 RAs (2.08). Also, higher proportions were observed for ALB (2.85), EXE (2.72), and LIX (2.08). Conversely, for LIR and DUL, other very prevalent GLP-1 RAs, the proportion of ADRs from the total ICSRs was lower (1.81, respectively 1.90). However, for TIR the proportion (1.98) was similar to SEM (Figure 5).

According to data published in EV, ADRs reported in the group of patients aged 18–64 years treated with SEM (39.7%) had a close frequency with all other GLP-1 RAs (41.8%), ALB (42.2%) and DUL (37.9%). Also, in the 65–85 years group, no great differences have been observed for SEM (21.6%) compared to the group of all other analogues (22.7%) and ALB (18.8%) (Table 3).

The most frequent cases have been reported in the female group that used SEM (57.7%) compared to the group of all other GLP-1 RAs (53.6%). Also, a similar frequency can be observed for LIR (59.8%). The most reported cases from EEA were for SEM (52.6%), similar to DUL (51.1%), but more frequent than the entire group of all other GLP-1 RAs. Healthcare professionals have been the ones to most often report ADRs related to SEM (61.3%). The same situation was noticed for all other GLP-1 RAs, except ALB (45.6%). Regarding the severity, the cases reported in EV as serious represented 74.2% (n = 38,215) of the total number related to all GLP-1 RAs. It can be noticed that serious cases reported for SEM (n = 12,029; 57.2%) had the lowest frequency compared to all other analogues (Table 3).

#### 3.2.2. Comparative Evaluation of ADRs Grouped by SOC

**Figure 6:**
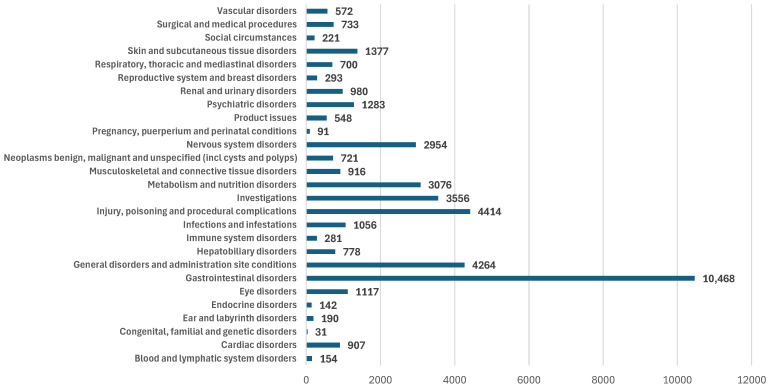
Distribution of ADRs reported for SEM by SOC.

Firstly, a comparison between the distribution of ADRs by SOC was conducted between SEM and all other GLP-1 RAs. Thus, it could be observed that the ADRs of SEM were most frequently reported in the following SOCs: “Gastrointestinal disorders” (25.0%; n = 10,468), “Injury, poisoning and procedural complications” (10.6%; n = 4414), “General disorders and administration site conditions” (10.2%; n = 4264). Similar situations were obtained for the ADRs reported in SOCs “Gastrointestinal disorders” and “General disorders and administration site conditions” for all comparators. Also, in the SOC “Injury, poisoning and procedural complications”, similar situations were observed for ALB (11.8%) and TIR (10.0%). Among the SOCs with the lowest ADR reporting frequency were: “Congenital, familial and genetic disorders”, “Pregnancy, puerperium and perinatal conditions”, and “Social circumstances” (Figure 6).

#### 3.2.3. ADRs Reported for Incorrect Dosage

**Figure 7:**
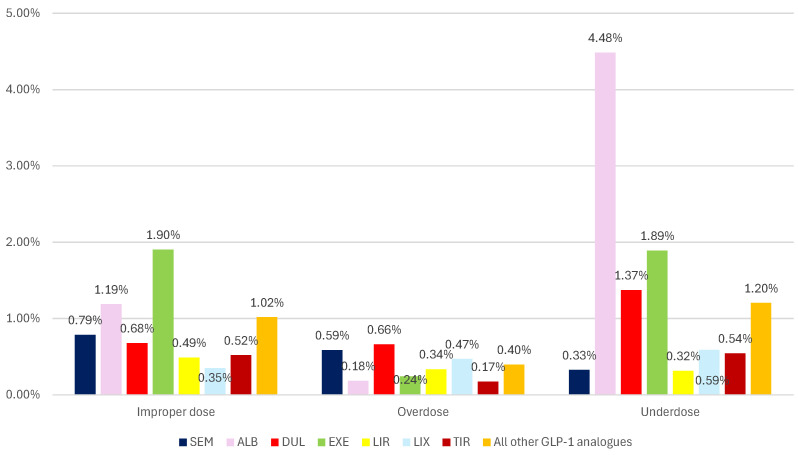
Frequency of ADRs related to dosage in GLP-1 RA series. ALB—albiglutide; DUL—dulaglutide; EXE—exenatide; LIR—liraglutide; LIX—lixisenatide; SEM—semaglutide; TIR—tirzepatide.

Figure 7 presents the frequency of ADRs related to dosage in GLP-1 RAs class. Thus, for SEM, the most frequent are ADRs related to improper dose (0.79%), a percentage higher than that of DUL (0.68%), TIR (0.52%), and LIR (0.49%), but lower than that of EXE (1.90%), and the entire group of all other analogues (1.02%). Overdoses have been reported for SEM (0.59%) with a lower frequency than for DUL (0.66%), but higher than those reported for all other comparators. Underdoses have the lowest frequency (0.33%) in HLTs related to the dosage of SEM. This percentage is similar to LIR (0.32%) and inferior to all other comparators.

#### 3.2.4. ADRs Reported as “Off-Label Use”

**Figure 8:**
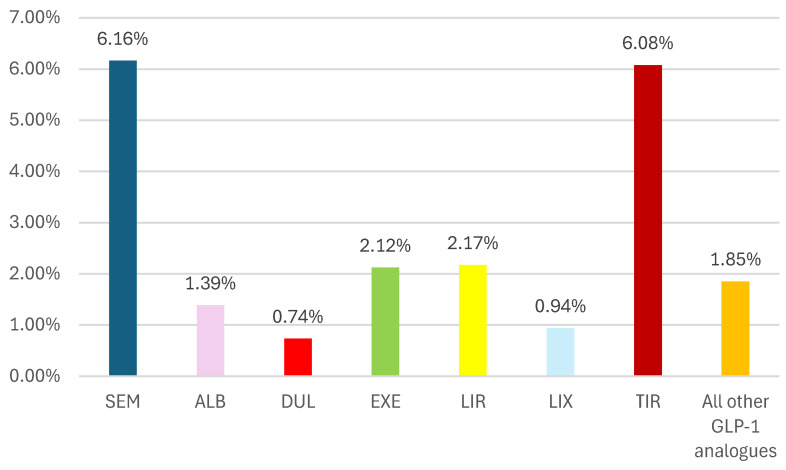
Frequency of ADRs related to off-label use in GLP-1 RA series. ALB—albiglutide; DUL—dulaglutide; EXE—exenatide; LIR—liraglutide; LIX—lixisenatide; SEM—semaglutide; TIR—tirzepatide.

According to Figure 8, for the ADRs reported for off-label use, their frequency in the SEM series (6.16%) is similar to TIR (6.08%), but higher than other comparators, including the entire group of all other GLP-1 RAs.

#### 3.2.5. Distribution of ADRs by Outcome

##### ADRs Reported for SEM

**Figure 9:**
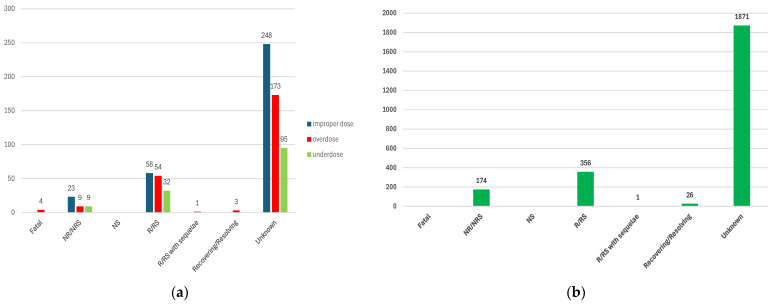
Distribution of ADRs of semaglutide by outcome: (**a**) related to dosage; (**b**) related to off-label use. NR/NRS—not recovered/not resolved; NS—not specified; R/RS—recovered/resolved; ALB—albiglutide; DUL—dulaglutide; EXE—exenatide; LIR—liraglutide; LIX—lixisenatide; SEM—semaglutide; TIR—tirzepatide.

Regarding the outcomes, Figure 9 shows that unfavorable outcomes were reported as follows: (i) 4 cases related to overdoses were fatal; (ii) 41 cases related to incorrect dosage were not recovered or not resolved (23 for improper doses; 9 for overdosage, 9 for under dosage, respectively); (iii) 356 cases related to off-label use were not recovered or not resolved.

##### The Frequency of ADRs with Unfavorable Outcomes Reported for SEM Compared to All Other GLP-1 RAs

**Figure 10:**
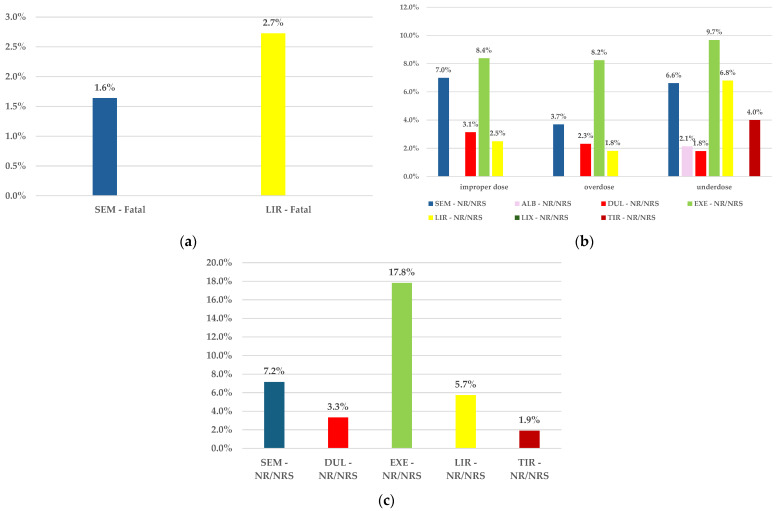
The frequency of ADRs with unfavorable outcomes. (**a**) Fatal outcomes of ADRs related to incorrect dosage; (**b**) not recovered/not resolved outcomes of ADRs related to incorrect dosage; (**c**) not recovered/not resolved outcomes of ADRs related to off-label use. NR/NRS—not recovered/not resolved; NS—not specified; R/RS—recovered/resolved; ALB—albiglutide; DUL—dulaglutide; EXE—exenatide; LIR—liraglutide; LIX—lixisenatide; SEM—semaglutide; TIR—tirzepatide.

The unfavorable outcomes of cases reported in EV (fatal or not recovered/not resolved) are represented below. Thus, Figure 10a presents the frequency of fatal ADRs reported for incorrect dosage. Fatal ADRs were reported only for overdosage, with a higher frequency in the LIR series (2.7%) than for SEM (1.6%). Also, for off-label use, no fatal ADRs were reported for any GLP-1 RAs.

The frequency of the not recovered/not resolved outcomes is presented in Figure 10b. According to this data, SEM presented a higher frequency in all three HLTs, compared with all other GLP-1 RAs, with the following exceptions (Figure 10b):

- improper doses: SEM—7.0% and EXE—8.4%
- overdose: SEM—3.7% and EXE—8.2%
- underdose: SEM—6.6%, EXE—9.7%, and LIR—6.8%

Regarding the frequency of the not recovered/not resolved ADRs related to off-label use, SEM (7.2%) also had a higher frequency of being reported compared to other GLP-1 RAs, except EXE (17.8%) (Figure 10c).

### 3.3. Disproportionality Analysis

#### 3.3.1. Incorrect Doses

**Figure 11:**
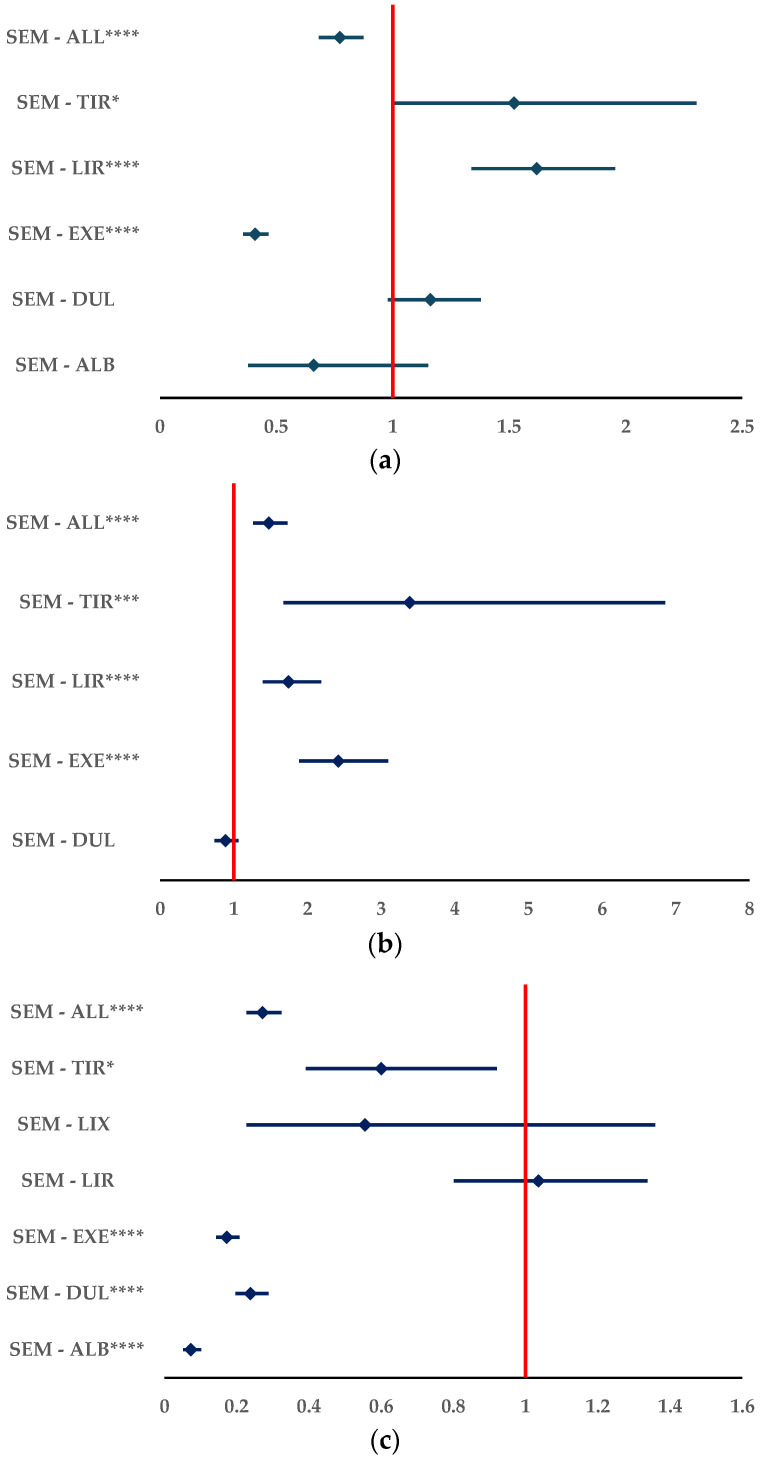
Disproportionality analysis for incorrect dosage of SEM: (**a**) improper dosage; (**b**) overdose; (**c**) underdose. ALB—albiglutide; DUL—dulaglutide; EXE—exenatide; LIR—liraglutide; LIX—lixisenatide; SEM—semaglutide; TIR—tirzepatide. * *p* <0.05; *** *p* ≤ 0.001; **** *p* ≤ 0.0001.

According to the data published in EV, the results of the disproportionality analysis show a higher probability of reporting ADRs related to improper doses for SEM compared to LIR (ROR: 1.6169, 95% CI: 1.3379–1.9542) and TIR (ROR: 1.5200, 95% CI: 1.0032–2.3031). Also, in Figure 11a, a lower probability of ADRs for SEM compared to EXE (ROR: 0.4087, 95% CI: 0.3579–0.4667) and the entire group of all other GLP-1 RAs (ROR: 0.7722, 95% CI: 0.6823–0.8740) could be observed. No difference could be observed for SEM compared to DUL and ALB.

For ADRs related to overdose, SEM had a higher probability of being reported compared to EXE (ROR: 2.4174, 95% CI: 1.8878–3.0956), LIR (ROR: 1.7434, 95% CI: 1.3913–2.1845), and TIR (ROR: 3.3868, 95% CI: 1.6736–6.8535). The same results could be noticed through a comparison with the group of all other GLP-1 RAs (ROR: 1.4764, 95% CI: 1.2608–1.7288). Also, when compared to DUL, no difference could be noticed (Figure 11b).

For overdosage, SEM had a higher probability of being reported, but for underdosage the situation is reversed. Thus, no difference could be observed by comparison with LIR and LIX, but a lower probability of being reported could be observed by comparison with all other analogues and with the entire group of all other GLP-1 RAs (Figure 11c).

#### 3.3.2. Off-Label Use

**Figure 12:**
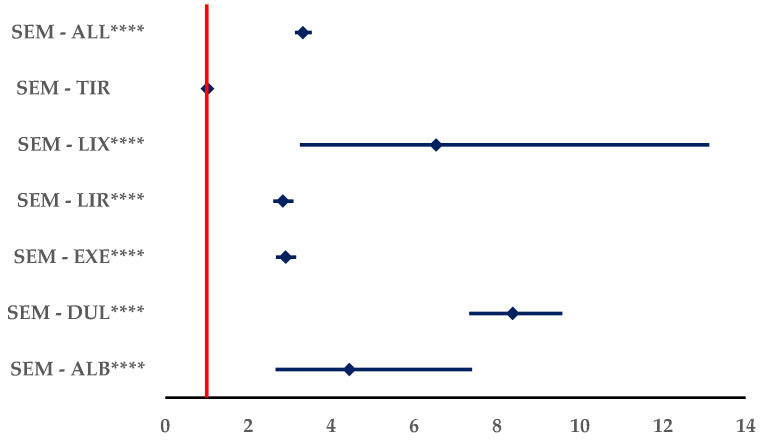
Disproportionality analysis for off-label use of SEM. ALB—albiglutide; DUL—dulaglutide; EXE—exenatide; LIR—liraglutide; LIX—lixisenatide; SEM—semaglutide; TIR—tirzepatide. **** *p* ≤ 0.0001.

Figure 12 showed a higher probability of reporting off-label use for SEM compared to all other analogues, except TIR: ALB (ROR: 4.4375, 95% CI: 2.6615–7.3988), DUL (ROR: 8.3830, 95% CI: 7.3359–9.5796), EXE (ROR: 2.9018, 95% CI: 2.6680–3.1560), LIR (ROR: 2.8377, 95% CI: 2.6052–3.0910), and LIX (ROR: 6.5330, 95% CI: 3.2525–13.1221). Also, compared to the group of all other GLP-1 RAs the probability of being reported is higher (ROR: 3.3226; 95% CI: 3.1270–3.5304).

## 4. Discussion

A reduced appetite and food intake were observed after the administration of GLP-1 RAs. Thus, their benefits in weight loss are exploited by using them in obese patients with or without diabetes [70]. Improving the patients’ adherence to GLP-1 RAs treatment through possible oral administration [71] was an important objective of the researchers following their approval on the market. Only a few years after the EXE approval in therapy, new molecules have been authorized. SEM presents some differences in pharmacokinetics due to the modifications in GLP-1 structure: (i) improved stability against dipeptide-peptidase-4 enzyme (DPP-4) through the substitution of alanine with aminoisobutyric acid; (ii) increased binding to albumin by the introduction of a linker and a C18 di-acid chain; (iii) preventing the binding of fatty acid at the wrong site through the substitution of Lys with Arg [72]. On the other hand, to improve the patients’ adherence to SEM, its absorption across gastric mucosa was improved by obtaining a co-formulation with sodium N-(8-[2-hydroxybenzoyl]amino)caprylate. Based on this formulation, SEM was the first of the GLP-1 RAs suitable for oral administration [73]. Thus, SEM was expected to be very popular in the media and widely used in therapy, often as off-label or as auto medication. Because of this issue, the dosing errors were expected to be quite frequent.

The first item analyzed was the popularity of SEM in Google searches. The term “weight loss semaglutide” was the most frequently searched for by the people who also searched for the term “semaglutide”. Also, the queries related to weight loss have a higher frequency than for the other GLP-1 RAs. Additionally, the search interest on Google for each molecule was related to a lower frequency with the side effects (Table 2). Based on this information, it could be considered that the people interested in these molecules had similar search behavior regarding the safety of the products. Our results are comparable to the ones in the study performed by Han et al., which showed that the greatest relative search was obtained for one of the SEM brand names [7]. Also, a study published in 2024 showed that SEM was one of the most popular pharmacological and surgical obesity methods searched on Google [74]. The popularity of SEM on TikTok (an online social media platform), is also very high. A total of 57 of the first 100 video searches under the hashtag “#Ozempic” were related to “weight loss” (44 million views), and 29 of 100 were related to “common side effects, toxicity” (24 million views). The “off-label” use was a search term only in 3 of 100 videos (2.8 million views) [75].

Although SEM was approved on the market more recently than LIR, DUL, EXE, and ALB, the descriptive analysis showed the highest number of ICSRs reported in EV (29% of the total) (Figure 4). Its popularity (Figure 2) and indication in obesity could contribute to an increase in the prescription numbers, as well as implicitly in its consumption. In 2022, the global market of SEM and LIR increased by 43% from 9.9 to 14.2 billion USD [76]. Moreover, according to a study published in 2023, prescriptions for SEM increased by 150%/year [77]. This increase could be justified by a higher efficiency of SEM in weight loss and by a lower cost of treatment [78,79]. Other studies showed the superior efficiency of SEM compared to LIX [80], EXE [80,81], DUL [81] and, LIR [82]. For example, SEM led to an average reduction in body weight of 12.4% in 68 weeks compared to LIR (−5.4% in 56 weeks) [82]. Therefore, SEM had an improved value for money in weight reduction compared with LIR (estimated cost of 1845 USD per 1% reduction in body weight for SEM, compared to 3256 USD for LIR) [82].

Regarding the demographic characteristics of patients, in the present study, most of the reports were in the 18–64 years group (Table 3). Also, the most frequent reports were for females (57.7%) (Table 3), similar to the results of another study performed on the data from the Food and Drug Administration Adverse Event Reporting System (FAERS) between 2018–2022 (54.4%) [9].

According to the present study, SEM had the lowest number of severe ADRs compared to the analyzed molecules (Table 3). These results are similar to other studies that reported a safety profile consistent with other GLP-1 RAs, with a low incidence of severe ADRs for SEM [81,83,84]. Generally, SEM is well tolerated, and most ADRs induced are mild-to-moderate and transient. The most frequent ADRs are related to the “Gastrointestinal disorders” SOC (nausea, vomiting, diarrhea, pancreatitis, etc.), or the “General disorders and administration site conditions” SOC (e.g., malaise). On the other hand, pathologies such as acute kidney injury from the “Renal and urinary disorders” SOC could have negative consequences on health status even if they are less frequent (Figure 6). Most probably, nausea and vomiting are caused by the inhibition of gastric emptying, and diarrhea could be induced by altering nutrient absorption or intestinal motility [83]. Additionally, malaise seems to be promoted by direct central GLP-1 R activation, primarily in the brainstem [85]. Regarding pancreatitis and pancreatic cancer, the FDA and EMA concluded that no causal association could be found between GLP-1 RAs and these pathologies. Only a few preclinical studies have shown a pancreatic inflammatory status after GLP-1 RA use [83,86]. Dehydration caused by nausea, vomiting or diarrhea, as well as increasing the sodium excretion after GLP-1 RA administration, could lead to renal failure [83,87]. To diminish gastrointestinal disturbances, different strategies could be applied: gradual dose titration, eating slowly, reducing the portion size per meal, avoiding high-fat food, and finishing eating before satiety. Also, it is recommended to avoid the risk factors for renal failure, such as dehydration or association with medication with a high renal risk [83].

Fatal outcomes were only reported for overdosing on SEM and LIR. Anyway, until now, no unexpected safety issues have been reported for SEM [83,84]. However, another interesting result of the present study suggests that SEM had a higher number of ADRs reported by each case than DUL and LIR, and was similar to those of TIR (Figure 5). Considering that TIR was recently authorized, it is expected that this situation will be different over time.

This study revealed a higher tendency to report ADRs related to overdosing of SEM (except DUL and EXE), and incorrect dosing compared to LIR and TIR (Figure 7). According to the American Association of Poison Control Centers, a total of 2941 cases related to SEM overdosing were reported between January–November 2023, more than double compared to 2022 [88]. The overdosing cases reported in the scientific literature were associated with notable gastrointestinal symptoms, and even with medical evaluation and treatment with antiemetics and intravenous fluids [89,90].

On the other hand, the off-label use of SEM was more frequently reported in EV than other GLP-1 RAs (except TIR) (Figure 8). Its advantages (high efficiency and safety, improved value for money in weight reduction, and increased benefits–risk ratio) probably represent factors for increasing the off-label use of SEM. According to the study performed by Chiappini et al., the off-label use of SEM was the fifth most frequent cause of reporting in FAERS (6%) [9].

Finally, media attention fuels the demand for this type of medication and, at the same time, generates an increase in illegal sales. Thus, the authorities in countries such as Austria, Denmark, United Kingdom, Ireland, Switzerland, etc., seek to repress illegal activity with these drugs, approaching different methods of social media monitoring, even reporting the confiscation of falsified pens with SEM in some EEA states [91]. Moreover, both manufacturers and regulatory agencies in the field of medicine issued warnings about the penetration of counterfeit products into the drug supply chain, finding them in retail pharmacies [92,93,94]. The warnings were issued by reglementary authorities such as the FDA, EMA, and the Medicines and Healthcare Products Regulatory Agency from the United Kingdom. These refer to using the unapproved salt forms of SEM or to online sales of fraudulent or unapproved products [95,96,97]. In this context, greater attention must be paid, both to the way of prescribing and also to the counselling of patients regarding the identification of fakes and the judicious use of medicines.

### Limitations of the Study

Certain limitations should be considered for this study. Our searches in Google Trends only included active pharmaceutical ingredients. The online environment is extensive; besides Google, other search engines and social media platforms are widely used. We acknowledge that these results may not offer the full depiction of the off-label use for weight loss phenomenon. On the other hand, the use of the Internet, including the Google search engine, is reduced in different areas with limited access to internet or freedom of speech. Also, people with lower socioeconomic status or educational background, or old people, represent categories with low access to computers or internet [56,59]. Other limitations of this study are based on the analysis of ADRs from the EudraVigilance spontaneous reporting system, among which some are related to the phenomenon of underreporting, overreporting and reporting bias or to the inaccuracy of the information contained in the reports. The number of ADRs reported could be influenced by the extent of drug use, the awareness of the reporter, media coverage of the drug, the severity and outcome of the reaction, and the variability of reporting rates between different regions, etc. ADRs associated with newer drugs or severe cases might be reported more frequently compared with older drugs or minor adverse effects. Not least, off-label use could be a factor in the underreporting of adverse drug reactions. Moreover, information such as concomitant medication or other suspected drugs, comorbidities, medical status, etc., could be missing, thus affecting this analysis. Other limitations are the lack of a denominator or the lack of certainty of a causal relationship between the reported ADRs and the suspected drug. Furthermore, the ROR is a simple indicator that allows for the estimation of the relative risk of ADR reporting but could not be used to quantify the true risk. Further studies are needed for an extensive evaluation of the safety profile of SEM and other GLP-1 RAs.

## 5. Conclusions

Our study highlights the risk of the improper or off-label use of SEM based on an analysis of real-world data from Google Trends and the European spontaneous reporting system, EudraVigilance. To reduce these risks, especially of severe ADRs or unfavorable outcomes, stakeholders should promote the correct use and dispensing of drugs based on SEM. Also, an increased carefulness in patient counselling could improve healthcare outcomes. Likewise, new studies must be performed to obtain information regarding dosing errors or off-label use. Based on the results obtained following the analysis of ICSRs, useful information can be provided for a better monitoring and managing of adverse events related to abuse. Taking into consideration the limitations of spontaneous reporting of ADRs, a high level of standardization or more detailed reporting could improve the quality of data and strengthen the robustness of future analyses.

## Figures and Tables

**Table 1:** The preferred terms used for analysis of ICSRs uploaded in EudraVigilance.

HLT	PT
modified dose	Dose calculation error
	Dose calculation error associated with device *
	Drug dose titration not performed
	Drug titration error
	Incorrect dosage administered
	Incorrect dose administered
	Incorrect dose administered by device
	Incorrect dose administered by product
	Incorrect product dosage form administered *
	Product dosage form confusion *
	Wrong dose
overdose	Accidental overdose
	Intentional overdose
	Extra dose administered
	Overdose
	Prescribed overdose
underdose	Accidental underdose
	Drug dose omission by device
	Incomplete dose administered *
	Intentional dose omission
	Intentional underdose
	Prescribed underdose
	Product dose omission *
	Product dose omission in error
	Product dose omission issue
	Underdose
Off-label use	Contraindicated product administered
	Contraindicated product prescribed
	Drug effective for unapproved indication *
	Off-label use
	Off-label use of device
	Product use in unapproved therapeutic environment *
	Product use in unapproved indication
	Product used for unknown indication *
	Unintentional use for unapproved indication

* PT with no reports.

**Table 2:** Comparison between the most frequent queries related to “side effects” and “weight loss” [69].

	Terms Associated with “Weight Loss”	Terms Associated with “Side Effects”
SEM	weight loss semaglutide	side effects semaglutide
	semaglutide for weight loss	
	ozempic weight loss	
LIR	liraglutide weight loss	liraglutide side effects
	liraglutide for weight loss	
TIR	tirzepatide weight loss	tirzepatide side effects
	tirzepatide for weight loss	
	semaglutide weight loss	
DUL	dulaglutide weight loss	dulaglutide side effects
EXE	exenatide weight loss	exenatide side effects

**Table 3:** Characteristics of ICSRs reported for SEM. EEA—European Economic Area; HP—healthcare professional; NS—not specified; ALB—albiglutide; DUL—dulaglutide; EXE—exenatide; LIR—liraglutide; LIX—lixisenatide; SEM—semaglutide; TIR—tirzepatide.

	SEM	ALB	DUL	EXE	LIR	LIX	TIR	All Other GLP-1 RAs
	n (%)	n (%)	n (%)	n (%)	n (%)	n (%)	n (%)	n (%)
Total	21,012	384	17,332	12,926	18,149	412	2333	51,536
Age category								
NS	7942	148	5476	4160	6668	105	1138	17,695
	(37.8)	(38.5)	(31.6)	(32.2)	(36.7)	(25.5)	(48.8)	(34.3)
0–1 Month	1	0	1	6	4	0	0	11
	(0.0)	(0.0)	(0.0)	(0.0)	(0.0)	(0.0)	(0.0)	(0.0)
2 Months–2 Years	2	0	3	1	4	0	0	8
	(0.0)	(0.0)	(0.0)	(0.0)	(0.0)	(0.0)	(0.0)	(0.0)
3–11 Years	9	0	2	1	9	0	0	12
	(0.0)	(0.0)	(0.0)	(0.0)	(0.0)	(0.0)	(0.0)	(0.0)
12–17 Years	23	0	6	6	64	0	2	78
	(0.1)	(0.0)	(0.0)	(0.0)	(0.4)	(0.0)	(0.0)	(0.2)
18–64 Years	8345	162	6576	5519	8236	186	863	21,542
	(39.7)	(42.2)	(37.9)	(42.7)	(45.4)	(45.1)	(37.0)	(41.8)
65–85 Years	4546	72	4951	3153	3104	118	307	11,705
	(21.6)	(18.8)	(28.6)	(24.4)	(17.1)	(28.6)	(13.2)	(22.7)
More than 85 Years	144	2	317	80	60	3	23	485
	(0.7)	(0.5)	(1.8)	(0.6)	(0.3)	(0.7)	(1.0)	(0.9)
Sex								
Female	12,122	211	8443	6741	10,851	210	1180	27,636
	(57.7)	(54.9)	(48.7)	(52.2)	(59.8)	(51.0)	(50.6)	(53.6)
Male	8206	158	7676	5825	6189	169	685	20,702
	(39.1)	(41.1)	(44.3)	(45.1)	(34.1)	(41.0)	(29.4)	(40.2)
NS	684	15	1213	360	1109	33	468	3198
	(3.3)	(3.9)	(7.0)	(2.8)	(6.1)	(8.0)	(20.1)	(6.2)
Geographic origin								
EEA	11,060	12	8864	3226	7309	265	115	19,791
	(52.6)	(3.1)	(51.1)	(25.0)	(40.3)	(64.3)	(4.9)	(38.4)
NON-EEA	9952	372	8468	9700	10,839	147	2218	31,744
	(47.4)	(96.9)	(48.9)	(75.0)	(59.7)	(35.7)	(95.1)	(61.6)
NS	0	0	0	0	1	0	0	1
	(0.0)	(0.0)	(0.0)	(0.0)	(0.0)	(0.0)	(0.0)	(0.0)
Reporter								
HP	12,877	175	11,001	8751	12,316	333	1470	34,046
	(61.3)	(45.6)	(63.5)	(67.7)	(67.9)	(80.8)	(63.0)	(66.1)
NHP	8135	209	6331	4168	5832	79	863	17,482
	(38.7)	(54.4)	(36.5)	(32.2)	(32.1)	(19.2)	(37.0)	(33.9)
NS	0	0	0	7	1	0	0	8
	(0.0)	(0.0)	(0.0)	(0.1)	(0.0)	(0.0)	(0.0)	(0.0)
Seriousness								
Non serious	8983	5	7051	1383	4637	119	120	13,315
	(42.8)	(1.3)	(40.7)	(10.7)	(25.5)	(28.9)	(5.1)	(25.8)
NS	0	0	0	3	3	0	0	6
	(0.0)	(0.0)	(0.0)	(0.0)	(0.0)	(0.0)	(0.0)	(0.0)
Serious	12,029	379	10,281	11,540	13,509	293	2213	38,215
	(57.2)	(98.7)	(59.3)	(89.3)	(74.4)	(71.1)	(94.9)	(74.2)

## Data Availability

Data contained within the article.
